# Socioeconomic Variation in Emotional, Cognitive, and Behavioural Engagement with the Climate Crisis in England: Perspectives for Education

**DOI:** 10.3390/bs15040407

**Published:** 2025-03-23

**Authors:** Rachael C. Edwards, Joy Perry, Nicola Walshe

**Affiliations:** Centre for Climate Change and Sustainability Education, Institute of Education, University College London, 20 Bedford Way, London WC1H 0AL, UK; rachael.edwards@ucl.ac.uk (R.C.E.); j.perry.15@ucl.ac.uk (J.P.)

**Keywords:** climate change and sustainability education, climate crisis, climate justice, constructive hope, eco-anxiety, pro-environmental behaviour, socioeconomic inequalities, student perspectives

## Abstract

Issues of economic inequality are inextricably linked to the present climate and environmental crisis, with disadvantaged groups facing disproportionate impacts. This paper explores the intersection of socioeconomic status (SES) and degrees of emotional, cognitive, and behavioural engagement with the climate crisis, as well as equity in the provision of climate change and sustainability education (CCSE). We surveyed over 2000 students (ages 11–14) in England, comparing responses between students with the most (*n* = 599) and fewest (*n* = 389) books at home (as a proxy for SES). Students from lower socioeconomic backgrounds were significantly less worried about a climate-altered future, had lower levels of knowledge about climate change, and were less likely to undertake a range of pro-environmental behaviours. Our findings also emphasise the critical role of the school environment in promoting engagement with the climate crisis and the need to improve provision of CCSE for disadvantaged groups. Further, they reveal severe socioeconomic inequalities in the perceived experiences of CCSE and participation in climate and sustainability action, which was observed even when these activities were made available. This suggests that school systems must consider other possible explanations for whether disadvantaged children and young people engage with these issues beyond their opportunity to do so.

## 1. Introduction

Particularly over the last few decades, climate change has brought about a marked increase in extreme weather events, habitat and biodiversity loss, and associated economic, health, and wellbeing impacts (IPCC, 2022). Given the rate and severity of climate change and its impacts, it is unsurprising that there has been a corresponding rise in associated emotions, including worry, fear, grief, and anger (Léger-Goodes et al., 2022; Newlove-Delgado et al., 2023). Collectively, these emotional reactions to climate change and environmental degradation have been conceptualised as eco-anxiety (Pihkala, 2020). Through a nation-wide survey of students (ages 11–14) in England, we examine the complex relationship between socioeconomic status and emotional, cognitive, and behavioural engagement with our planet’s climate emergency. We explore these topics in the context of the provision of climate and sustainability education in the UK.

### 1.1. Responses to the Climate Crisis: Eco-Anxiety and Pro-Environmental Behaviour

Eco-anxiety is experienced particularly acutely among children and young people (Boluda-Verdú et al., 2022; Hickman et al., 2021). For example, a recent survey of 3000 children (aged 12–18) in the UK found that 70% worried about the world they will inherit (Save the Children, 2022). Given the prevalence of eco-anxiety, a growing body of literature has sought to identify educational strategies to address it. For example, the UK Royal College of Psychiatrists (2023a) and the UNESCO-supported Office for Climate Education (2024) have published resources for parents and teachers, respectively, to support young people with eco-anxiety. This literature widely recommends an emotionally responsive and action-orientated educational approach that provides space for young people to voice their concerns and develop their sense of agency (Chawla, 2020; Trott, 2022).

Eco-anxiety also has a role to play in addressing the climate crisis through motivating pro-environmental behaviour. Indeed, many scholars and educators have described eco-anxiety as a natural response to the climate crisis and that experiencing these emotions is necessary to inspire the drastic societal change that needs to occur (Comtesse et al., 2021; Galway et al., 2021; Ojala et al., 2021). For example, Skilling et al. (2022, p. 658) contend that “certain forms of hope are barriers to meaningful action, and that anxiety and despair are valid and potentially productive responses to reality”. This argument is supported by the growing body of evidence demonstrating a positive association between experiences of eco-anxiety and pro-environmental behaviour (e.g., Bouman et al., 2020; Pavani et al., 2023). This link is, however, complex, and the literature is only beginning to unpack its mediating factors (Brosch & Steg, 2021; Edwards et al., 2024; Pihkala, 2020). For example, Ojala and Bengtsson (2019)’s work suggests that behavioural responses to eco-anxiety will depend on one’s engagement in problem-focused coping, as opposed to de-emphasising the issue. As such, scholars have advocated for a balanced educational approach which acknowledges the scale of the problem and necessary change but also instils action-orientated hope to avoid despair and disengagement—this has been termed constructive or critical hope (Chawla, 2020; Ojala, 2016).

### 1.2. Socioeconomic Inequalities and Correlations with Eco-Anxiety

Given the impacts of eco-anxiety on wellbeing and its links to pro-environmental behaviour, it is critical that we enhance our understanding of its prevalence and severity across the population (Aylward et al., 2022; Whitmarsh et al., 2022). In this research, we are interested in the relationships between the socioeconomic status (SES) of children and young people and their experiences of eco-anxiety and pro-environmental behaviour. We selected this topic given that those of lower SES will be disproportionately impacted by climate change (Shonkoff et al., 2011), which will further exacerbate health inequalities (Royal College of Paediatrics and Child Health, 2023). For example, climate vulnerability reports show that deprived areas in the UK are subject to higher flood and heat risks (UK Health Security Agency, 2024). And yet, those experiencing poverty have contributed the least to the climate crisis both nationally and internationally, with consumption of the world’s bottom 50% of carbon emitters contributing a mere tenth of global emissions (Bruckner et al., 2022). Considering these inequalities, understanding how eco-anxiety is experienced by economically disadvantaged young people is necessary to inform more targeted educational strategies that offer appropriate support and strengthen their sense of agency, participation in climate activism, and influence on climate policy and practice.

Much of the literature exploring the relationship between SES and eco-anxiety is relatively recent, and results have been highly mixed (van der Linden, 2017), with little research focused on young people. For example, through a nation-wide survey of Australian adults, Patrick et al. (2023) identified higher rates of eco-anxiety in more disadvantaged regions. Conversely, Vercammen et al. (2023) found moderate levels of climate distress among 16–24-year-olds in the UK to be more prevalent in higher SES brackets. Other survey-based research in the UK found no relationship between climate anxiety and income (Whitmarsh et al., 2022). This variation is likely due in part to the diversity of methods and contexts in which these studies have been undertaken.

The literature on SES and eco-anxiety contains theoretical perspectives supporting both a positive and negative relationship. For example, scholars have drawn upon Inglehart (1990)’s theory of post-materialism to explain a positive association between SES and eco-anxiety. This framework postulates that as people gain wealth and have their basic needs met, greater concern can be afforded to wider issues such as climate change. A case study in a low-income suburb in Sydney lends support to this theory, finding that heatwaves were perceived as a “luxury problem” and downgraded to a secondary issue compared with poverty and housing needs (Zografos et al., 2016). On the other hand, scholars have made a distinction between the attention afforded to climate change and perceptions of risk to explain negative associations between SES and eco-anxiety (e.g., Lo & Chow, 2015). In other words, while those of greater affluence may believe more strongly in the seriousness of climate change, they do not perceive it to be as much of a personal threat compared to those with less adaptive resources.

The varying theories and mixed results on the relationship between SES and eco-anxiety indicate that further context-specific exploration is needed, particularly among young people. Inequalities in educational opportunities, in particular, could mediate socioeconomic differences in experiences of eco-anxiety as higher educational attainment can bring about greater awareness of environmental issues. For example, through a survey in 25 European countries, Niedzwiedz and Katikireddi (2023) found a positive association between education attainment and worry about climate change and that income was not associated with climate worry once education was accounted for. Drawing on this work, but focusing on a more specific setting, the present study explores the relationship between eco-anxiety, climate knowledge, pro-environmental behaviour, and SES in the context of the provision of climate and sustainability education (CCSE) in the UK.

### 1.3. The Role of Climate Change and Sustainability Education in Fostering Emotional, Cognitive, and Behavioural Engagement with the Climate Crisis

It is widely acknowledged that high-quality CCSE is pivotal to ensuring younger generations are prepared to navigate the climate and environmental crisis (Reid, 2019). Broadly speaking, provisions for ‘climate change education’ encompass human and natural causes of changing climate patterns, their scientific processes, and the impacts to society and the environment (UNESCO, 2016). Its counterpart, ‘sustainability education’, refers to more specific interactions between social, economic, and environmental systems, and whether human activity ‘meets the needs of the present without compromising the ability of future generations to meet their own needs’ (World Commission on Environment and Development, 1987). In theory, these two notions complement and inform one another to encourage critical thinking and innovative problem solving, propelling students to actively and constructively engage with the issues at hand. Schools have a central role to play in the provision of CCSE, equipping young people with the knowledge, skills, and capabilities to behave as global citizens and support Earth’s livelihood (Morrissey Gleeson & Morrissey, 2024).

Our education systems, if sufficiently mobilised, can stimulate effective climate change responses, spearheading the necessary societal transformation (Greer et al., 2024; Nusche et al., 2024). This is in part because principles of CCSE, in addition to cultivating knowledge and understanding of the crisis, reveal its accompanying inequalities. When evaluated as part of education’s greater interdisciplinary purposes, CCSE can inform the sector’s duties to safeguard healthy and equitable outcomes for children across the socioeconomic spectrum. Unfortunately, favourable socioeconomic circumstances tend to align with greater educational opportunity, and while this advantage often accrues over time, positive social mobility typically compounds with other disparities of gender, ethnicity, and disability (Cattan et al., 2022; Marmot et al., 2020; Shaw et al., 2016). For example, a recent study of UK households found that a person’s educational level positively correlated with their climate-related knowledge, with higher-educated cohorts more likely to support climate policy and have lower carbon footprints (Hampton & Whitmarsh, 2024). Inequalities in access to CCSE could thus directly contribute to SES variation in eco-anxiety and pro-environmental behaviour.

The pedagogical approaches adopted within CCSE are critical to fostering constructive emotional responses. For example, Finnegan (2023)’s survey of students and teachers in the UK showcases a positive correlation between climate change-related ‘hope’ and ‘action competence’ and revealed the teaching practices that best predict student-reported hope. These findings suggest that pedagogical tone, as well as content, contribute to students’ behavioural engagement in the climate crisis. Indeed, it is critical that CCSE resonates with students in ways which engage them in ‘head, hands, and heart’ (i.e., cognitively, behaviourally, and emotionally) (Sipos et al., 2008; UNESCO, 2020). Such is a hallmark of what Glackin and Greer (2025) refer to as meaningful transformative education in a period of environmental and social crisis: one which embraces the complexities of these issues and channels multiple worldviews and educational approaches to bring about real-world systemic change. Our study adds to this educational discourse by evaluating the interrelated processes of thinking, feeling, and acting through which students convey responses to the climate crisis and socioeconomic variance among them.

### 1.4. Research Context: Climate Change and Sustainability Education in England

In England, schools are only mandated to teach climate change per the subject of secondary science, while there is no explicit mention of sustainability (Department for Education, 2014). To improve upon such inadequacies, the Department for Education (2022) introduced a Sustainability and Climate Change Strategy which included new guidelines for sustainable school practices. While this strategy is at face value a victory for proponents of CCSE, its language continues to uphold science as the primary subject through which it should be delivered. This dismisses the interdisciplinary nature of the topic and why multiple school subjects are necessary for unpacking its complexities and activating interest and engagement (Greer et al., 2024). Labelled a ‘placebo policy’, the new strategy also offers little guidance to teachers who are reportedly ill-equipped to successfully carry out CCSE (Dunlop & Rushton, 2022), risking the readiness of England’s younger generations to meet the demands of the climate emergency (Greer et al., 2023).

Due to its loose interpretation and seemingly over-reliance on individually motivated and capable school personnel, the current approach to CCSE arguably widens educational inequalities, an inherently antithetical outcome to its presumed purposes. Positively, the Department for Education (2024) recently launched an independent Curriculum and Assessment Review, inviting stakeholder discourse concerning curricular strengths and weaknesses, including those pertaining to CCSE. This government commission introduces a possibility for positive educational reform and more holistic assimilation of CCSE into English school systems. The present study is thus timely, using a breadth of student experiences to forefront a more refined and equitable approach to CCSE.

### 1.5. Research Questions and Approach

This study draws on data collected as part of a nation-wide survey of Year 7–9 students (ages 11–14) in England conducted by the University College London Centre for Climate Change and Sustainability Education (Walshe et al., 2024). The survey sought to identify *what* and *how* students are currently learning about climate change and sustainability, including their concerns and aspirations for the future. The survey aimed to amplify student voices on these issues, a key component to CCSE-related research which is currently underexplored yet vital for informing pedagogical practices and identifying inequalities. The survey builds on prior reports that students feel inadequately prepared to lead sustainable lives or apply practical knowledge in real-world situations (Wheeler, 2023) and desire an up-to-speed curriculum which enables them to tackle socioeconomic and political issues affecting their immediate settings and communities (British Science Association, 2023).

We examined a selection of survey data concerned with eco-anxiety, climate knowledge, pro-environmental behaviours, and the provision of CCSE in the context of SES. In essence, we use students’ lived experiences as a catalyst for understanding the social justice and civic engagement dimensions of climate change. Homing in on socioeconomic disparities in relation to CCSE is, we argue, crucial for exposing systemic issues, empowering learners, and promoting inclusive solutions.

To explore the intersection of these issues, we ask the following questions:

Research question 1 (RQ1): How are children and young people’s emotional, cognitive, and behavioural responses towards the climate and environmental crisis distinguished by SES?

Research question 2 (RQ2): What do lived experiences of students reveal about opportunities for CCSE in connection with SES?

We use these findings to prompt further discussion about possible provisions for ensuring equitable access to high-quality CCSE in England, irrespective of SES, and how schools can support disadvantaged students in developing pro-environmental attitudes and behaviours towards these issues.

## 2. Materials and Methods

This research involved a nation-wide survey of Year 7–9 students (ages 11–14) in England. We applied a holistic approach, exploring and integrating a range of the survey data to answer each of our research questions.

### 2.1. Survey Instrument

The survey was designed to capture a wide range of information on student experiences and perspectives of climate change and sustainability; it was developed using a collaborative, iterative process of drafting, team discussion and testing, followed by final amendments in response to a pilot study with one class of Year 8 students (*n* = 32). The survey questions were designed with consideration of prior literature and national surveys (e.g., Cheng & Monroe, 2012; Hunt et al., 2017; Lereya et al., 2016; Natural England, 2023) and ranged in style and content to keep participants engaged with the process, as well as to glean a breadth of data for understanding their responses. The survey was designed to be completed in 30–40 min using Qualtrics (see Section 2.2). In addition to demographic information, data from seven of the survey questions that aligned with our research questions were analysed for this paper (Supplementary Materials Figure S1).

Prior to data collection, we obtained ethical approval from the Institutional Research Ethics Committee and the informed consent of each participant. Because teachers distributed the survey, students were made explicitly aware that there was no school-related benefit or consequence to their participation and that doing so was entirely of their own volition. Data were managed securely according to UK GDPR and DPA 2018 guidelines and were anonymised before the analysis stage.

The initial section of the survey encompassed key demographic questions. We gathered data on students’ school year group, gender, ethnicity, and SES. Like leading educational surveys (e.g., Martin et al., 2019), the latter was not measured directly but evaluated in terms of the number of books or eBooks at home. This is because, in England, the number of books at home has been inversely correlated with eligibility for free school meals; the more books a student has at home, the less likely they are to qualify for free school meals (Richardson et al., 2020). Use of this metric, ranging from ‘*None to very few (0–10 books)*’ to ‘*Enough to fill three or more bookcases (more than 200)*’, allowed for key cross-analysis of data and is likely indicative of socioeconomic inequality.

We explored the concept of eco-anxiety by asking students whether they felt a range of 16 emotions relating to climate change through a Yes or No format. ‘Negative’ (e.g., anxiety, anger, despair), ‘positive’ (e.g., optimism, happiness), and ‘neutral’ (i.e., uninterested) emotions were included. As another indicator of eco-anxiety, we also asked students if they worried about what the world would be like in the future because of climate change using a four-point Likert scale from “*Not at all*” to “*All the time*”. Climate knowledge was captured through asking students their extent of agreement with six statements relating to climate change (three accurate statements and three inaccurate ones) on a five-point Likert scale from “*Strongly agree*” to “*Strongly disagree*”.

We captured engagement in pro-environmental behaviour through asking students if they “*Always*”, “*Sometimes*”, or “*Never”* undertook a range of thirteen behaviours, including turning off the lights, recycling, and talking to friends and family about looking after the environment.

Three questions were used to explore experiences of CCSE. Students were asked to indicate whether they had learnt about climate change and/or sustainability through five channels (both within and external to the school environment) using a Yes/No format. Students were also asked if they agreed with seven statements relating to the quality of CCSE they had received using a five-point Likert scale from “*Strongly agree*” to “*Strongly disagree*”. These included questions relating to students’ perceived ability to influence the school environment and share their experiences/views, clarity in teaching about climate concepts, and enjoyment and interest in learning about these topics. Finally, students were asked if they had participated in a range of ten school-based and extra-curricular climate change/sustainability activities. To distinguish the availability of these activities from participation, students were given three options when asked if they had had the opportunity to take part in each activity: “*Yes, and I HAVE taken part*”, “*Yes, but I have not taken part*”, and “*No*”.

### 2.2. Recruitment and Survey Administration

We recruited young people in Years 7 to 9 (those aged 11 to 14) in England between March and May 2024. The recruitment process was carried out with the help of teachers who were contacted electronically through a variety of networking mediums, including invitations issued through our faculty and research group distribution lists, social media channels, and e-newsletters. The recruitment activity aimed to reach students from all geographical regions and socioeconomic backgrounds across England, although most responses were ultimately gathered from schools in London and the south–east of England. The survey was explained and administered by teachers in school, either as part of regular lessons or form time. Teachers were given detailed guidelines for administering the surveys; instructions for how to complete the surveys were also available for students as part of the survey preamble, alongside the participant information and consent process.

### 2.3. Sample

We received responses from 2429 students from 30 schools across England (24 state-funded and six independent), although not all students answered every question. Twenty-five percent of our sample comprised students with the most books at home (*n* = 599), while students with the fewest books at home made up 16 percent (*n* = 389) (Table 1). There was between 30 and 40% representation from all three-year groups among both those with the most and fewest books, apart from Year 9 respondents with the most books, who comprised only 24% of the sample. While respondents with the fewest books at home were relatively evenly split between girls and boys (52% and 47%, respectively), girls made up a much larger proportion of the respondents with the most books (58%). One percent of the sample in both socioeconomic groups comprised non-binary respondents. The sample of students with the most books at home was comprised predominantly of students from white backgrounds (70%). Conversely, only 49% of students with the fewest books at home described themselves as white. Among students with the most and fewest books, Asian/Asian British were the second most represented ethnic groups (16% and 25%, respectively). No other ethnic group made up more than 10% of the sample among students with the most or fewest books.

### 2.4. Analysis

For this article, all our analyses revolve around comparing responses from students with the most (more than 200) and fewest (0–10) books at home. We selected this approach to offer an accessible and efficient exploration of the SES ‘gap’ among students, which is highly meaningful in the context of the current CCSE policy and practice landscape. We explored differences in climate emotions, climate knowledge, pro-environmental behaviours, and CCSE experiences between these groups through independent-samples *t*-tests (without assuming equal variances across the groups of students being considered). Magnitudes of difference were considered through D values, which can be interpreted through values below 0.20 reflecting a minimal difference, values from 0.20 to 0.50 reflecting a small difference, values from 0.50 to 0.80 reflecting a moderate difference, and values above 0.80 reflecting a large difference (Cohen, 1988).

## 3. Results

Our results are presented such that Section 3.1 and Section 3.2 address RQ1: How are children and young people’s emotional, cognitive, and behavioural responses towards the climate and environmental crisis distinguished by SES? Section 3.3 addresses RQ2: What do lived experiences of students reveal about opportunities for CCSE in connection with SES?

### 3.1. Emotional and Cognitive Responses to Climate Change

In response to the statement ‘*I worry about what the world will be like in the future because of climate change*’, 36% of all students indicated that they worried ‘*a lot*’ or ‘*all the time*’. The extent to which students worried varied significantly based on socioeconomic background. Only 20% of students with the fewest books at home reported feeling worried ‘*a lot*’ or ‘*all the time*’ compared to 47% of students with the most books (d = −0.591, *p* < 0.001).

**Figure 1:**
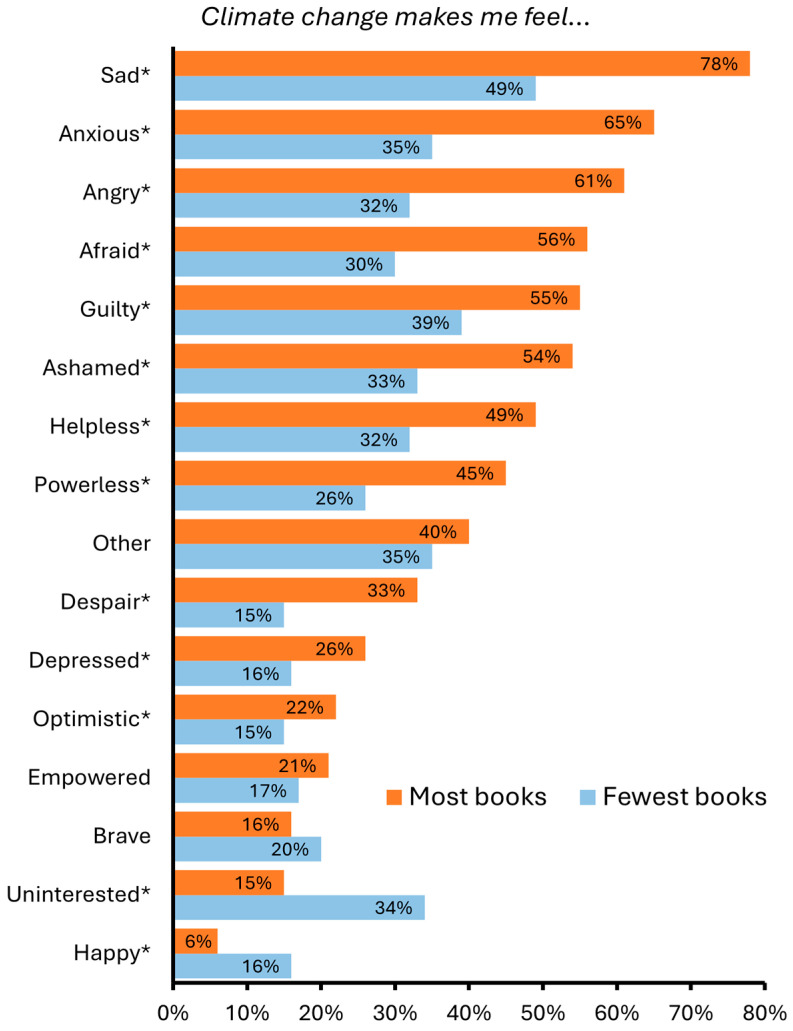
Percentage of students with the most and fewest books at home who conveyed various emotions relating to climate change (percentages of those selecting ‘*Yes*’ for each emotion in response to the statement ‘*Climate change makes me feel ….*’). Significant differences are indicated with an asterisk (*).

Similar trends emerged when students were asked whether they experienced a range of sixteen emotions in response to the statement ‘*Climate change makes me feel…*’. Relatively small numbers of students expressed feeling ‘positive’ emotions such as optimism compared to ‘negative’ emotions like guilt and fear. There were significant differences between those with the fewest and most books at home for all but three of the emotions (one of which was ‘*other*’) (Figure 1, Supplementary Materials Table S1). Eleven of the emotions were significantly more prevalent among students with the most books. For example, 78% of students with the most books at home indicated that they felt sad about climate change compared with only 49% of students with the fewest books at home. While most of these emotions were ‘negative’, students with the most books at home were also more likely to feel optimistic. However, students with the fewest books at home were more likely to indicate they felt happy and uninterested in climate change.

**Figure 2:**
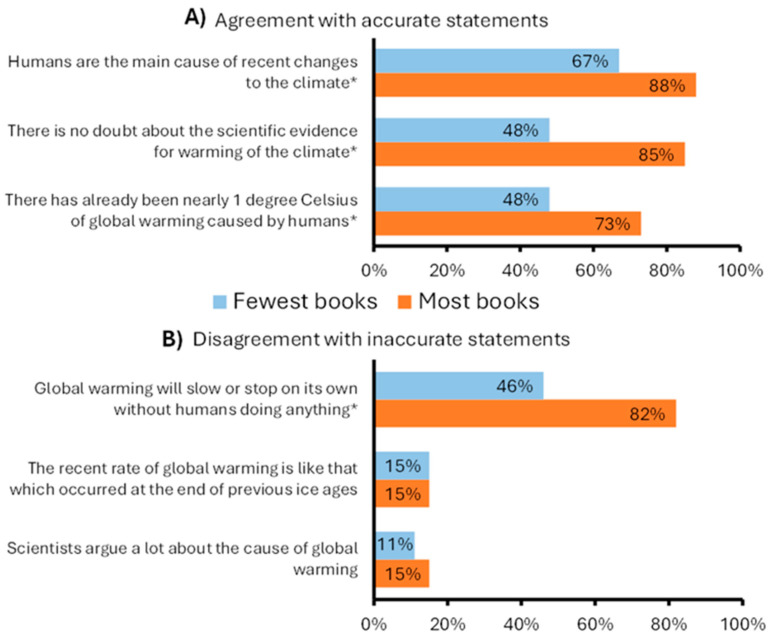
Percentage of students with the most and fewest books at home who agreed with accurate statements. (**A**) (‘*Strongly agreed*’ or ‘*Agreed*’ rather than ‘*Neither agree nor disagree*’, ‘*Disagree*’, or ‘*Strongly disagree*’) and disagreed with inaccurate statements. (**B**) (‘*Strongly disagree*’ or ‘*Disagree*’ rather than ‘*Neither agree nor disagree*’, ‘*Agree*’, or ‘*Strongly agree*’) about climate change. Significant differences are indicated with an asterisk (*).

Our results also reveal significant socioeconomic differences in climate knowledge. When asked about their extent of agreement with a variety of accurate and inaccurate statements about climate change, responses from students with the fewest books were significantly less likely to align with the correct answer than students with the most books for four of six statements (Figure 2, Supplementary Materials Table S2). In all four cases, the magnitude of the difference was moderate to large. For example, only 46% of students with the fewest books at home “*Strongly disagreed*” or “*Disagreed*” with the statement “*Global warming will slow or stop on its own without humans doing anything*” compared with 82% of students with the most books. Among the students with the fewest books at home, the percentage who conveyed ambivalence (selecting ‘*Neither agree nor disagree*’) was high (ranging from 23 to 58%). As such, in many cases, these students might not have disagreed with accurate statements (or agreed with inaccurate ones) but rather have been unsure. For students with the most books at home, the proportion selecting ‘*Neither agree nor disagree*’ was much lower for all but two statements.

### 3.2. Engagement in Pro-Environmental Behaviour

**Figure 3:**
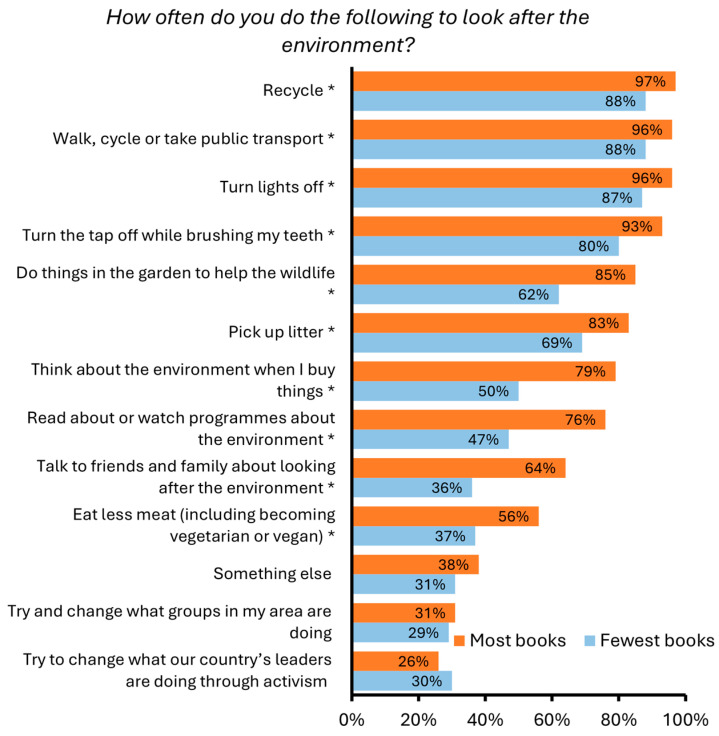
Percentage of students with the most and fewest books at home who indicated that they ‘*Always*’ or ‘*Sometimes*’ (rather than ‘*Never*’) performed a range of pro-environmental behaviours. Significant differences are indicated with an asterisk (*).

Significant socioeconomic differences emerged in relation to students’ engagement in pro-environmental behaviours. Engagement in ten of the thirteen pro-environmental behaviours in the survey varied significantly between students with the most and fewest books. Students possessing the most books undertook all ten behaviours more often (Figure 3, Supplementary Materials Table S3). For example, 79% of students with the most books at home ‘*Always*’ or ‘*Sometimes*’ thought about the environment when buying things compared to 50% of students with the fewest books at home. When compared to engagement in day-to-day pro-environmental behaviours, an overall smaller number of students indicated that they had tried to change what the country’s leaders are doing through activism or what groups are doing in the area. There were also no differences in engagement in these behaviours between students with the most and fewest books.

### 3.3. Experiences and Perspectives of Climate Change and Sustainability Education

**Figure 4:**
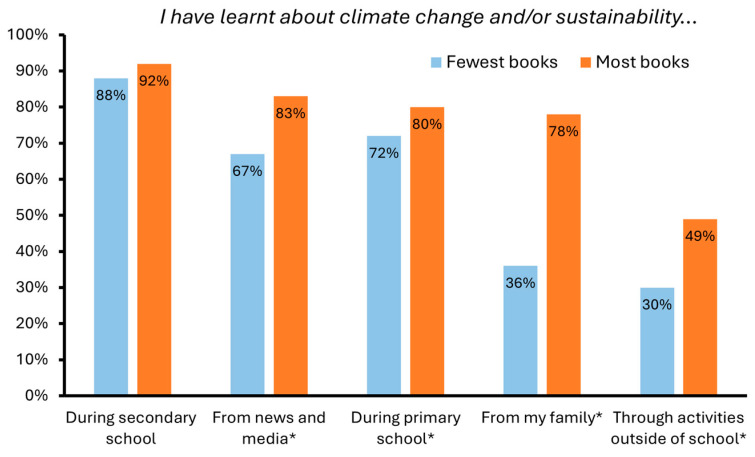
Percentage of students with the most and fewest books at home who indicated that they learnt about climate change and/or sustainability through various channels (percentages of those selecting ‘*yes*’ for each learning opportunity in response to the statement ‘*I have learnt about climate change and/or sustainability…*’). Significant differences are indicated with an asterisk (*).

Our results reveal significant socioeconomic differences in students’ experiences of CCSE. Students with the most books at home were significantly more likely to have learnt about climate/sustainability from their families, through activities outside school, and from news and media (Figure 4, Supplementary Materials Table S4). Although this same divide was observed in primary school, the magnitude of the difference was smaller, and students with the most and fewest books were equally likely to have learnt about climate change/sustainability during secondary school.

**Figure 5:**
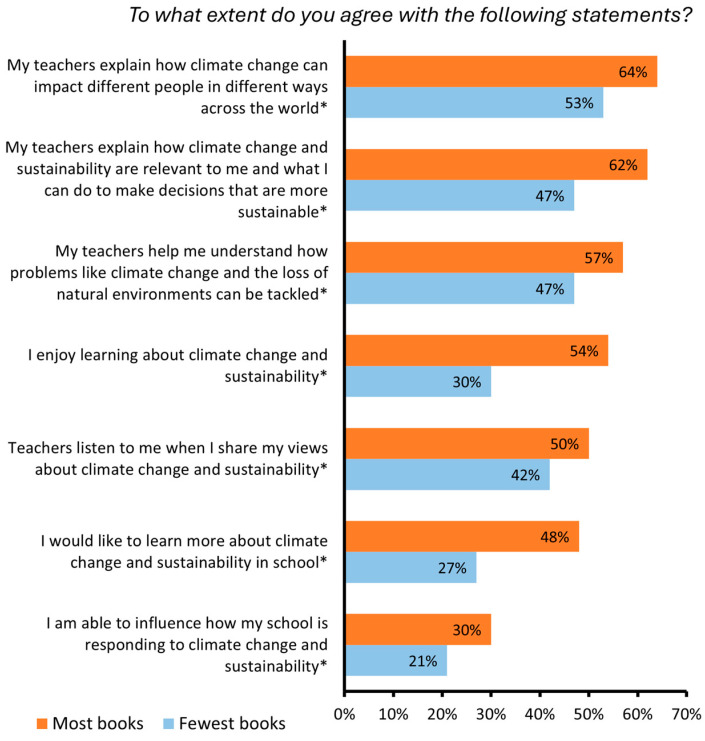
Percentage of students with the most and fewest books at home who indicated that they ‘*Agree*’ or ‘*Strongly agree*’ (rather than ‘*Neither agree nor disagree’*, ‘*Disagree*’, or ‘*Strongly disagree*’) with a range of statements about their climate/sustainability education. Significant differences are indicated with an asterisk (*).

There were also significant socioeconomic differences in student perceptions of their climate/sustainability education (Figure 5, Supplementary Materials Table S5). Compared to students with the most books at home, students with the fewest books at home were significantly less likely to ‘*Agree*’ or ‘*Strongly agree*’ with all seven statements relating to their climate/sustainability education, including about the clarity of their teacher’s explanation of concepts, actions that can be taken, opportunity to share their views, and opportunity to influence their school. For example, 50% of students with the most books at home felt that their teachers listened to their views about climate change/sustainability compared with 42% of students with the fewest books.

**Figure 6:**
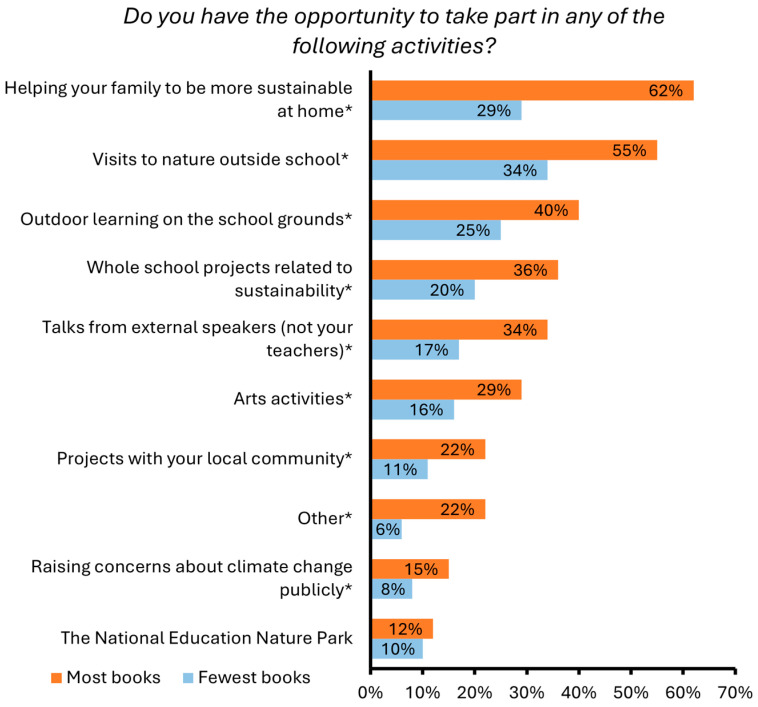
Percentage of students with the most and fewest books at home who indicated that ‘*Yes, and I HAVE taken part*’ in a range of activities in relation to climate change and/or sustainability (rather than ‘*Yes, but I HAVE NOT taken part*’ or ‘*No*’). Significant differences are indicated with an asterisk (*).

There were also significant socioeconomic differences in students’ participation in a range of ten school-based and extra-curricular climate change and/or sustainability activities (Figure 6, Supplementary Materials Table S6). Significantly more students with the most books at home participated in all but one of these activities (the National Education Nature Park). For example, while 40% of students with the most books at home engaged in outdoor learning on the school grounds, only 25% of students with the fewest books at home took part in this activity. An even wider divide in participation can be seen relating to helping one’s family be more sustainable, in which 62% and 29% of students with the most and least books took part, respectively.

To further understand socioeconomic differences in participation in climate/sustainability activities, we explored both differences in availability of these opportunities and participation in the opportunities when they were available. We identified significant socioeconomic differences in the availability of six of the climate/sustainability activities: students with the most books at home had better access to all six opportunities compared to those with the fewest books (Supplementary Materials Table S7). The greatest difference was in the availability of opportunities to help one’s family be more sustainable at home (this opportunity was available to 76% of students with the most books compared to only 51% of students with the fewest books). However, the magnitude of the difference was small in the case of all other significant differences.

We observed significant socioeconomic differences in students’ participation in all climate/sustainability activities when these activities were available, and the magnitudes of difference were larger than those relating to variance in the availability of these activities (Supplementary Materials Table S8). Students with the most books at home were more likely to participate in all cases. The greatest differences related to students’ participation in helping their families be more sustainable at home, visiting nature outside of school, and attending talks from external speakers. For example, only 57% of students with the fewest books at home visited nature outside school when they had the opportunity compared to 80% of students with the most books at home.

These findings suggest that participation in climate/sustainability activities is more influenced by the personal choice of whether to participate than by the availability of these activities. In other words, equal access to climate/sustainability activities does not, on its own, guarantee equal participation; rather, the socioeconomic disparity in participation is largely influenced by the decision-making of different groups. This suggests that understanding *why* (or why not) individuals engage in climate/sustainability activities is just as significant as understanding *when* or *how* they engage. In addition to opportunity, the former likely depends on factors including knowledge and awareness, personal values and biases, time or responsibility constraints, and sociocultural influences.

## 4. Discussion

Our findings suggest that the SES of young people in England relates to their experiences of eco-anxiety, climate knowledge, engagement with pro-environmental behaviour, and receipt of quality CCSE and wider opportunities to engage in climate/sustainability activities. In the following sections, we summarise our findings relating to socioeconomic variation in emotional, cognitive, and behavioural responses (Section 4.1) and the provision of CCSE (Section 4.2), threading associated implications for CCSE throughout.

### 4.1. Socioeconomic Variation in Emotional, Cognitive, and Behavioural Engagement with Climate Change

Sadness was the most frequently reported emotion relating to climate change across all students, as well as among students with the most and fewest books. Nevertheless, students from lower SES backgrounds demonstrated less worry towards, and emotional engagement in, the climate crisis. A wide range of ‘negative’ emotions, including sadness, anger and guilt, as well as optimism, were each less prevalent among those with the fewest books at home. Conversely, these students were more likely to report feeling ‘happy’ when asked about climate change, as well as being ‘uninterested’ in the topic. It is important not to assume that these students necessarily *derive* happiness from climate change but to consider this particular response, alongside disinterest, as indicative of their overall emotional detachment.

Existing literature on the relationship between SES and eco-anxiety is both under-researched and mixed. Our results align with Vercammen et al. (2023), Berry and Peel (2015), and others who identified a positive relationship between SES and eco-anxiety. As previously discussed, for many families living in deprived circumstances, climate change is one of many challenges they experience on a day-to-day basis, which could reduce the concern it is afforded (Inglehart, 1990); this could partially explain the relationship identified in this study. The context might also present an explanation for why our findings contradict the idea that individuals from lower SES backgrounds will experience a higher perception of individual climate risk (e.g., Lo & Chow, 2015). While countries such as the USA and Australia have experienced a growing number of devastating environmental disasters, the UK has yet to undergo climate-induced severe weather events of this scale (although they are predicted to occur). Although lower SES communities are at increased climate risk (UK Health Security Agency, 2024), the relatively moderate impacts they have experienced may contribute to reduced perceptions of risk in comparison to other countries. For example, even individuals in the age group most vulnerable to heat have been found to have a low perception of heat health risk in the UK (Wolf et al., 2009). Further research is needed to understand climate risk perceptions among young people in the UK.

Our finding that knowledge about climate change also positively varied with SES further explains the higher prevalence of climate distress among more affluent students. As with SES, findings on the relationship between education and concern about climate change have been mixed (van der Linden, 2017). However, some scholars contend that education is a better predictor of environmental concern than income (Niedzwiedz & Katikireddi, 2023; Pampel, 2014). For example, as Zafar and Ammara (2024, p. 8) describe, “Education can have an impact on individuals’ psychological attributes, potentially leading them to become more open to new ideas and values that they encounter”. Aligning with this hypothesis, we found that students of higher SES had more positive perceptions of, and opportunities to participate in, CCSE (discussed in Section 4.2). These findings, along with other UK-based research (e.g., Hampton & Whitmarsh, 2024), suggest that inequities in educational experiences (and subsequently the attainment of climate knowledge) contribute to the observed positive relationship between SES and eco-anxiety.

We also observed significant socioeconomic differences in student engagement with several pro-environmental behaviours. These results align with the widely identified relationship between eco-anxiety and pro-environmental behaviour (Anneser et al., 2024; Bouman et al., 2020; Pavani et al., 2023). Many of these behaviours, such as recycling, turning off the lights, and picking up litter, are seemingly basic enough that, in most cases, they will be minimally impacted by financial unaffordability. Other behaviours are likely to require more effort and favourable circumstances, such as gardening, buying environmentally friendly products, and discussing ways of caring about the environment with family and friends. As such, the lower engagement in pro-environmental behaviours observed among students from lower socioeconomic circumstances is likely to be both a result of resource availability (e.g., time, money) and less emotional engagement in the climate crisis. Many additional factors are also likely to be contributing to the uptake of pro-environmental behaviour given the complexity of knowledge and/or value-to-action gap in this context (Colombo et al., 2023).

These results highlight a need to emotionally engage disadvantaged youth in the climate crisis, a group which will be most affected by it and is a critical voice in tackling the issue. We consider it promising that, in developing a sense of personal connection to the issues, students are likely to positively alter their behaviour. What is more, routinely engaging in the smaller-scale pro-environmental behaviours used in this survey could help build longer-term habits for leading more sustainable lifestyles, as well as positively inform individuals’ value and identity formation (Linder et al., 2022). But if many children and young people are not experiencing significant emotional responses to matters of climate change and sustainability, schools must review and amend their CCSE frameworks accordingly and in ways which address both students’ affective and cognitive development. This includes the often challenging but essential issue of climate justice. Students should understand imbalances between groups who contribute to, and those impacted by, climate change and the forces at play. Feelings such as anger and injustice, while unpleasant, can stimulate meaningful conversations for dissecting the complexities of the crisis which their generation must learn to navigate.

Teachers must be equipped to facilitate emotionally responsive and action-oriented pedagogies which lend themselves to constructive hope, utilising informed and solution-based mechanisms which do not unnecessarily instigate despair or helplessness (Chawla, 2020; Trott, 2022). Such approaches might include engagement with the climate and nature crises through the arts, such as through arts-in-nature experiences (Walshe et al., 2022), and activities within schools that foster a sense of individual and collective agency for young people by building opportunities for meaningful action (Ojala et al., 2021). Attention must also be paid to how experiences of eco-anxiety among children and young people sit within the nation’s wider mental health epidemic and consequences for education. Professional bodies have sounded the alarm amidst increasing levels of depression and anxiety among this group (e.g., Children’s Commissioner for England, 2021; Royal College of Psychiatrists, 2023b), while research authorised by the National Healthcare Service (NHS) recently discovered that one in five individuals aged from 8 to 25 were likely to have a mental health disorder (Newlove-Delgado et al., 2023). Raising students’ awareness of climate change amidst these trends is also likely to present an emotionally challenging task for teachers, many of whom might themselves be experiencing eco-anxiety. Furthermore, as with most educational provisions, the extent to which such a CCSE framework is carried out successfully will depend on school leadership and resources and the availability of professional development for teachers. There are concrete and philosophical barriers to revising even the most advantaged school systems, and especially those catering to low SES communities with often already limited resources.

### 4.2. Addressing Socioeconomic Inequities in Experiences of Climate Change and Sustainability Education

Our findings relating to students’ experiences of CCSE suggest that schools are a critical place to engage disadvantaged youth in these topics. When compared to CCSE in formal schooling, significantly greater socioeconomic divides were observed outside the school environment. Students of lower SES were far less likely to have learnt about climate change and sustainability in a range of external settings, such as from family or news outlets. Similarly, by far the greatest socioeconomic divide among participation in climate/sustainability activities related to opportunities to help one’s family be more sustainable at home. These trends, while troubling, legitimise CCSE’s potential for promoting equity and wellbeing and how it can prompt education systems to bring about social changes for a healthier and more sustainable future. They highlight the importance of ensuring school-based CCSE learning opportunities reach young people from lower socioeconomic backgrounds. The fact that more advantaged students will likely have many learning opportunities from their families suggests that CCSE pedagogies and opportunities should be developed which engage disadvantaged students, particularly when resources are limited.

While this study highlights the potential for school systems to help identify and mitigate disparities in climate knowledge and emotional engagement, it also demonstrates that the perceived experiences of this education are inferior for less advantaged groups. Disadvantaged students were not only less interested in learning about these subjects but were also less likely to report positive perceptions of their teachers’ explanations of concepts, such as how they may be personally impacted by the climate crisis. As described in the previous section, they also reported less content knowledge. Other research has similarly found that interest in climate change was higher in more affluent areas of the country (Hamlyn et al., 2024). In our survey, students with the fewest books exercised less agency in influencing their school environment or sharing their experiences and ideas. They also experienced less enjoyment from learning about climate change and sustainability. This is contrary to research on educational enjoyment more broadly, which has found no differences based on social class (Stopforth et al., 2024); a comparison which suggests that enjoyment of CCSE relies on distinguishable influences not usually observed among less advantaged learners. Further research is, therefore, needed to unpack these disparities in engagement with, and perceived quality of, CCSE. Questions of *why* should challenge the education sector to re-examine notions of educational equality under the myriads of influences likely affecting CCSE experiences and to implement its pedagogical and systematic frameworks accordingly.

We also observed significant socioeconomic differences in students’ participation in a wide range of climate/sustainability activities, both within and external to the school environment; students from disadvantaged backgrounds were less likely to take part in almost every activity. Our study adds unique insight into this issue through disentangling *availability* of these opportunities from *participation* when they are available. This analysis revealed no socioeconomic differences or relatively weak significant differences in students’ reporting of the availability of most climate/sustainability activities (apart from helping one’s family be more sustainable). Conversely, strongly significant socioeconomic differences in participation were observed for all activities when they were available. Even when presented with the opportunity, for example, just over half of the students from the lowest SES group visited nature outside of school.

These results indicate that to enhance participation in climate/sustainability activities among disadvantaged youth, CCSE must go beyond ensuring the availability of these activities. The sector must adopt a more holistic conceptualisation of accessibility and consider the many other dimensions of these activities that impact participation; for instance, the content, the types of activities offered, the cost, the times at which they are offered, who delivers the activities, and other intersectional barriers impacting access. A good example of this is Wang et al. (2015), who, in response to the widespread use of geographic availability as the only measure of park access, developed a multidimensional framework conceptualising the physical and non-physical dimensions of perceived access to urban green space. Such frameworks should inspire developers of CCSE to think unconventionally, being open to new ideas which reflect the socioemotional nature of their subjects. For example, less than half of the surveyed students with the least books at home felt that their teachers explained how climate change and sustainability are relevant to them. CCSE must, therefore, be communicated in a way that is more personally meaningful to young people experiencing deprivation to enhance emotional engagement. One of the most significant opportunities to achieve this is to formally recognise the distinctive role of all school subjects in providing young people with a holistic understanding of climate change and sustainability. A multidisciplinary approach would help students consider the climate and ecological crisis in different ways, reflecting its wide-ranging implications on all aspects of life; this would allow students to learn about the broader aspects that interest and matter to them, creating more opportunities to reach those students who appear to be currently disengaged, thereby reducing inequalities in relation to CCSE and fostering a sense of agency and action for all young people (Walshe et al., 2024). Strengthening emotional connection to these issues in this way could subsequently enhance interest and participation in a wider range of activities.

### 4.3. Limitations and Recommendations for Future Research

This research was not without limitations. Firstly, pro-environmental behaviours were self-reported, and no actual behaviour was observed, and it is possible that the prevalence of discrepancies between self-reported and actual behaviours might vary between students with the most and fewest books due to norms, values, and related expectation bias. However, this is a common technique employed in climate research with children given the feasibility challenges associated with measuring behaviour directly (Liu & Green, 2024). Additionally, we merged responses across students who reported “*always*” or “*sometimes*” performing each behaviour, thereby comparing the percentage of students who undertook the behaviours at all rather than the extent to which they performed the behaviours. This approach is likely to have reduced the influence of misreported behaviours. Similarly, although our use of a yes/no question format to explore experiences of emotional responses to climate change lacks depth/versatility, it minimised the potential for variation in students’ perceptions of their own emotional reactions to have influenced our results. In other words, we compared whether students had experienced the emotion at all rather than asking them to gauge the scale of these emotions.

Second, while researchers aimed to recruit students with a breadth of experiences and perspectives in relation to CCSE, including through surveying entire classes or year groups, it is conceivable that the teachers who assisted in the recruitment and administering processes were themselves already subscribed to elements of CCSE and pursued students accordingly. In other words, the strength of CCSE in classrooms surveyed could have been higher than in the average classroom. Most survey responses were also gathered from students in London and the south–east of England, suggesting that our data are not nationally representative. More work is required to appropriately represent other regions across England and their respective demographics. Emotional reactions to climate change, as well as how young people experience the impacts of climate change, may vary between urban and rural contexts, for example. A wide variety of other regional differences have been identified in England, including values and education, which could influence factors explored in this research (Butt et al., 2022; Ofsed, 2015).

A third limitation was the use of the number of books in students’ homes as a proxy for SES, which would likely have been more accurately assessed through, for example, parental occupation or household income. However, we did not attempt to capture this information, as many students would not be able to accurately report on it with the level of detail required. Furthermore, as previously described, the number of books at home has been inversely correlated with eligibility for free school meals in England (Richardson et al., 2020).

Participants in this study were purposefully pooled from Key Stage 3 (Years 7–9), within which students are normally studying all standard National Curriculum subjects. Similar research with other groups, including primary school students and those studying for GCSE and A Level examinations, as well as students from Scotland, Wales, and Northern Ireland, would be of value, broadening our understanding of the student experience in relation to CCSE. Longitudinal studies on this topic would also be useful, as they could reveal how student perspectives evolve over time and to which elements of CCSE this applies.

There were numerous methods we could have applied to explore our relationships of interest. As the data comprised part of a larger study, we undertook a range of preliminary analyses and determined the focused approach described earlier (i.e., comparing responses between students with the most and fewest books) to address our research questions. However, other approaches present a complimentary perspective, and results from correlational analyses considering the entire sample broadly affirm the findings in the present work (Supplementary Materials Table S9). Future research could build on our work and explore additional correlations (e.g., investigating associations between emotions and behaviours and interactions with SES).

While this research considers various SES discrepancies in relation to CCSE, it is beyond its scope to identify each of these or their necessary interactions. For example, SES variation among perceived experiences of CCSE would need to be entirely isolated from the others, such as emotional response and climate knowledge, to determine a more specific relationship with pro-environmental behaviour. We acknowledge the complexities and limitations of exploring this topic through comprehensive survey responses and have made every effort to draw fair and sensible inferences from our data. Future research could pursue more precise questions on whether and how any areas of discrepancy should be prioritised and what education can do to mitigate the influence of SES.

Lastly, our findings would benefit from supplemental qualitative data. Incorporating mixed methods, such as focus groups and interviews, would be particularly helpful in exploring motivations behind participant responses. For example, why do less advantaged groups choose not to participate in climate/sustainability-related activities even when they are available? Who or what would inspire disadvantaged youth to express their views or act on environmental issues? What are the most significant challenges these youth feel in relation to learning CCSE-related topics? Addressing these questions was outside the scope of this study, and future qualitative, in-depth exploration is needed to deepen our understanding of the sentimental, behavioural, and experiential disparities discussed above.

## 5. Conclusions

One of the challenges of today’s climate and environmental crisis is understanding socioeconomic variance in how individuals connect and engage with these issues. This is particularly apparent among children and young people, as gathered in our survey of nearly 2500 English students. There is a complex yet inextricable relationship between eco-anxiety and pro-environmental behaviour and probable consequences for how individuals learn about and meaningfully participate to combat issues of climate change and sustainability. Addressing these disparities is necessary not only for promoting equity and wellbeing but also for the sake of adequately equipping younger generations with the tools they need amidst this global emergency. Our research serves to inform the education sector specifically, including how best to engage disadvantaged youth in CCSE, mitigate disparities in climate knowledge and understanding, improve the perceived quality of CCSE among disadvantaged students, promote participation in climate/sustainability activities which extends beyond accessibility, and strengthen these individuals’ constructive sentiments towards issues of climate change and sustainability. By confronting these implications in earnest, school systems can contribute to a more sustainable, equitable, and healthier future for our most vulnerable populations.

## Figures and Tables

**Table 1:** Demographic overview of student respondents with the most and fewest books at home.

Demographic Characteristics		Most Books at Home (*n* = 599)	Fewest Books at Home (*n* = 389)
School Year	Year 7	39%	30%
	Year 8	37%	37%
	Year 9	24%	33%
Gender	Girls	58%	52%
	Boys	41%	47%
	Non-binary	1%	1%
Ethnicity	White	70%	49%
	Asian/Asian British	16%	25%
	Black/Black British	2%	8%
	Arab	1%	9%
	Mixed	8%	4%
	Other	3%	4%

## Data Availability

All analysed and and discussed data are presented in tables in the Supplementary Materials. The raw data are available upon request from the corresponding author due to privacy restrictions. Because this dataset contains the sensitive information of vulnerable groups, the authors need to ensure that those who access it comply with UCL’s data protection policies as well as our ethical research procedures.

## Supplementary Materials

The following supporting information can be downloaded at: https://www.mdpi.com/article/10.3390/bs15040407/s1. Figure S1: Survey Questions; Table S1: Percentage of students who conveyed various emotions (those who answered ‘*yes*’) in response to the question ‘*Climate change makes me feel …*’; Table S2: Percentage of students who indicated that they agreed (‘*Strongly agreed*’ or ‘*Agreed*’ rather than ‘*Neither agree nor disagree*’, ‘*Disagree*’, or ‘*Strongly disagree*’) or disagreed (‘*Strongly disagree*’ or ‘*Disagree*’ rather than ‘*Neither agree nor disagree*’, ‘*Agree*’, or ‘*Strongly agree*’) with six statements relating to climate change; Table S3: Percentage of students who indicated that they ‘*Always*’ or ‘*Sometimes*’ (rather than ‘*Never*’) performed a range of pro-environmental behaviours; Table S4: Percentage of students who indicated that they learnt about climate change and/or sustainability through various channels (those who answered ‘*yes*’ in response to the question ‘*I have learnt about climate change and/or sustainability…*’); Table S5: Percentage of students who indicated that they ‘*Agree*’ or ‘*Strongly agree*’ (rather than ‘*Neither agree nor disagree’*, ‘*Disagree*’, or ‘*Strongly disagree*’) to seven statements relating to their climate/sustainability education; Table S6: Percentage of students with the most and fewest books at home who indicated that ‘*Yes, and I HAVE taken part*’ to the question ‘*Do you have the opportunity to take part in any of the following activities in relation to climate change and/or sustainability?*’ (rather than ‘*Yes, but I HAVE NOT taken part*’ or ‘*No*’); Table S7: Percentage of students with the most and fewest books at home who indicated ‘*Yes, and I HAVE taken part*’ or ‘*Yes, but I HAVE NOT taken part*’ (rather than ‘*No*’) to the question ‘*Do you have the opportunity to take part in any of the following activities in relation to climate change and/or sustainability?*’; Table S8: Percentage of students with the most and fewest books at home who indicated ‘*Yes, and I HAVE taken part*’ (rather than ‘*Yes, but I HAVE NOT taken part*’) to the question ‘*Do you have the opportunity to take part in any of the following activities in relation to climate change and/or sustainability?*’ when activities were available; Table S9: Preliminary correlational analyses between students’ socioeconomic status and a range of measures capturing emotional, cognitive, and behavioural engagement with the climate crisis.
