# Supplementary material for: Socioeconomic Variation in Emotional, Cognitive, and Behavioural Engagement with the Climate Crisis in England: Perspectives for Education
Source: Behav Sci (Basel). 2025 Mar 23;15(4):407. doi: 10.3390/bs15040407 (PMC12024268; doi:10.3390/bs15040407)
Supplement: Supplementary file 1 [file behavsci-15-00407-s001.zip › behavsci-3509364-supplementary.pdf]

Supplementary materials

Figure S1: Survey questions

<u>Demographic information</u>

The first few questions are just so we know a little bit about you.

Q1. What is your school's name and town or city (e.g. St Mary's School in Leeds)?

Q2. Which school year are you in?

Year 7	
Year 8	
Year 9	

Q3. What best describes your gender?

Female	
Male	
Non-binary	
Prefer not to say	
Prefer to self-describe	

Q4. What best describes your ethnicity?

Arab	
Asian or Asian British (Bangladeshi)	
Asian or Asian British (Chinese)	
Asian or Asian British (Indian)	
Asian or Asian British (Pakistani)	
Asian or Asian British (other)	
Black, Black British, Caribbean or African (African)	
Black, Black British, Caribbean or African (Caribbean)	
Black, Black British, Caribbean or African (other)	
Mixed or Multiple ethnic groups (White and Asian)	
Mixed or Multiple ethnic groups (White and Black African)	
Mixed or Multiple ethnic groups (White and Black Caribbean)	
Mixed or Multiple ethnic groups (other)	
White (English, Welsh, Scottish, Northern Irish, or British)	
White (European)	
White (Gypsy or Irish Traveller)	
White (Irish)	
White (Roma)	

White (other)	
Prefer not to say	
Prefer to self-describe	

Q5. How many books or ebooks are there in your home? (Do not count magazines, newspapers, or your school books)

None or very few (0–10 books)	
Enough to fill one shelf (11–25 books)	
Enough to fill one bookcase (26–100 books)	
Enough to fill two bookcases (101–200 books)	
Enough to fill three or more bookcases (more than 200)	

Emotional response to climate change and climate knowledge

Q6. To what extent do you agree with the following statement

	Not at all	A little	A lot	All the time
I worry about what the world will be like in the future because of climate change				

Q7. Climate change makes me feel...

	Yes	No
Sad		
Happy		
Helpless		
Empowered		
Anxious		
Afraid		
Brave		
Angry		
Guilty		
Ashamed		
Depressed		
Despair		
Optimistic		
Powerless		
Uninterested		
Other		

Q8. To what extent do you agree with the following statements about climate change?

	Strongly agree	Agree	Neither agree nor disagree	Disagree	Strongly disagree
There is no doubt about the scientific evidence for warming of the climate					
Humans are the main cause of recent changes to the climate					
Scientists argue a lot about the cause of global warming					
There has already been nearly 1 degree Celsius of global warming caused by humans					
The recent rate of global warming is like that which occurred at the end of previous ice ages					
Global warming will slow or stop on its own without humans doing anything					

Pro-environmental behaviour

Q9. How often do you do the following to look after the environment?

	Always	Sometimes	Never
Eat less meat (including becoming vegetarian or vegan)			
Turn lights off			
Turn the tap off while brushing my teeth			
Think about the environment when I buy things (e.g. avoiding plastics)			
Recycle			
Do things in the garden to help the wildlife (e.g. feed birds, grow plants that insects like, make homes for wildlife)			
Pick up litter			
Walk, cycle or take public transport			
Read about or watch programmes about the environment			
Talk to friends and family about looking after the environment			
Try and change what groups in my area are doing e.g. by speaking to my scout leader or local council			

Try to change what our country's leaders are doing through activism			
Something else… (please add)			

Provision of climate and sustainability education

Q10. I have learnt about climate change and/or sustainability:

	Yes	No
During primary school		
During secondary school		
From news and media		
From my family		
Through doing activities outside of school, such as through clubs, volunteering, or School Strikes for Climate		

Q11. To what extent do you agree with the following statements

	Strongly agree	Agree	Neither agree nor disagree	Disagree	Strongly disagree
I am able to influence how my school is responding to climate change and sustainability					
Teachers listen to me when I share my views about climate change and sustainability					
My teachers explain how climate change and sustainability are relevant to me and what I can do to make decisions that are more sustainable					
My teachers explain how climate change can impact different people in different ways across the world					
My teachers help me understand how problems like climate change and the					

loss of natural environments can be tackled					
I would like to learn more about climate change and sustainability in school					
I enjoy learning about climate change and sustainability					

Q12. Do you have the opportunity to take part in any of the following activities in relation to climate change and/or sustainability?

	Yes, and I HAVE taken part	Yes, but I HAVE NOT taken part	No
Outdoor learning on the school grounds			
Talks from external speakers (not your teachers)			
Visits to nature outside school			
Arts activities			
The National Education Nature Park			
Projects with your local community			
Whole school projects related to sustainability			
Raising concerns about climate change publicly e.g., public speaking, protesting or writing to an MP			
Helping your family to be more sustainable at home			
Other			

Table S1:

Percentage of students who conveyed various emotions (those who answered ‘*yes*’) in response to the question ‘*Climate change makes me feel …*’. The results are presented across all students within the sample (‘All students’), and across groups of those with different extents of books at home (where ‘Fewest books’ refers to ‘None or very few (0–10 books)’ and ‘Most books’ refers to ‘Enough to fill three or more bookcases (more than 200)’), and the magnitude (‘D’; Cohen’s D) and statistical significance (‘Sig. (p)’; p-value) of the difference across those with different extents of books at home. Significant p-values (p < .05) and the associated magnitudes are highlighted in bold and shaded.

Climate change makes me feel …		Comparison<br>Percent who answered ‘*yes*’			
	All students	Most books	Fewest books	Cohen's d	Sig. (p)
Optimistic	19%	22%	15%	**.170**	**.015**
Happy	9%	6%	16%	**.347**	**<.001**
Empowered	18%	21%	17%	.090	.202
Brave	17%	16%	20%	.109	.139
Sad	67%	78%	49%	**.642**	**<.001**
Anxious	53%	65%	35%	**.620**	**<.001**
Guilty	50%	55%	39%	**.328**	**<.001**
Angry	48%	61%	32%	**.596**	**<.001**
Ashamed	47%	54%	33%	**.419**	**<.001**
Afraid	46%	56%	30%	**.529**	**<.001**
Helpless	42%	49%	32%	**.338**	**<.001**
Powerless	36%	45%	26%	**.382**	**<.001**
Despair	24%	33%	15%	**.415**	**<.001**
Depressed	20%	26%	16%	**.243**	**<.001**
Uninterested	23%	15%	34%	**.471**	**<.001**
Other	34%	40%	35%	.103	.259

Table S2:

Percentage of students who indicated that they agreed (‘*Strongly agreed*’ or ‘*Agreed*’ rather than ‘*Neither agree nor disagree*’, ‘*Disagree*’, or ‘*Strongly disagree*’) or disagreed (‘*Strongly disagree*’ or ‘*Disagree*’ rather than ‘*Neither agree nor disagree*’, ‘*Agree*’, or ‘*Strongly agree*’) with six statements relating to climate change. The results are presented across all students within the sample (‘All students’), and across groups of those with different extents of books at home (where ‘Fewest books’ refers to ‘None or very few (0–10 books)’ and ‘Most books’ refers to ‘Enough to fill three or more bookcases (more than 200)’), and the magnitude (‘D’; Cohen’s D) and statistical significance (‘Sig. (p)’; p-value) of the difference across those with different extents of books at home. Significant p-values ($p < .05$) and the associated magnitudes are highlighted in bold and shaded.

To what extent do you agree with the following statements about climate change?		Comparison			
	All students	Most books	Fewest books	Cohen's d	Sig. (p)
There is no doubt about the scientific evidence for warming of the climate **(% agreement)**	70%	85%	48%	**.890**	**<.001**
Humans are the main cause of recent changes to the climate **(% agreement)**	80%	88%	67%	**.535**	**<.001**
There has already been nearly 1 degree Celsius of global warming caused by humans **(% agreement)**	62%	73%	48%	**.539**	**<.001**
Scientists argue a lot about the cause of global warming **(% disagreement)**	11%	15%	11%	.100	.138
The recent rate of global warming is like that which occurred at the end of previous ice ages **(% disagreement)**	15%	15%	15%	.010	.890
Global warming will slow or stop on its own without humans doing anything **(% disagreement)**	65%	82%	46%	**.819**	**<.001**

Table S3:

Percentage of students who indicated that they '*Always*' or '*Sometimes*' (rather than '*Never*') performed a range of pro-environmental behaviours. The results are presented across all students within the sample ('All students'), and across groups of those with different extents of books at home (where 'Fewest books' refers to 'None or very few (0–10 books)' and 'Most books' refers to 'Enough to fill three or more bookcases (more than 200)'), and the magnitude ('D'; Cohen's D) and statistical significance ('Sig. (p)'; p-value) of the difference across those with different extents of books at home. Significant p-values (p < .05) and the associated magnitudes are highlighted in bold and shaded.

How often do you do the following to look after the environment?		Comparison<br>Percent who answered '*Always*' or '*Sometimes*' (rather than '*Never*')			
	All students	Most books	Fewest books	Cohen's d	Sig. (p)
Try to change what our country's leaders are doing through activism	29%	26%	30%	.076	.327
Try and change what groups in my area are doing	31%	31%	29%	.034	.660
Talk to friends and family about looking after the environment	51%	64%	36%	**.580**	**<.001**
Read about or watch programmes about the environment	61%	76%	47%	**.640**	**<.001**
Walk, cycle or take public transport	92%	96%	88%	**.282**	**.001**
Pick up litter	79%	83%	69%	**.341**	**<.001**
Do things in the garden to help the wildlife	76%	85%	62%	**.555**	**<.001**
Recycle	94%	97%	88%	**.346**	**<.001**
Think about the environment when I buy things	68%	79%	50%	**.662**	**<.001**
Turn the tap off while brushing my teeth	89%	93%	80%	**.409**	**<.001**
Turn off lights	93%	96%	87%	**.344**	**<.001**
Eat less meat (including becoming vegetarian or vegan)	44%	56%	37%	**.381**	**<.001**
Something else	32%	38%	31%	.157	.151

Table S4:

Percentage of students who indicated that they learnt about climate change and/or sustainability through various channels (those who answered ‘*yes*’ in response to the question ‘*I have learnt about climate change and/or sustainability…*’). The results are presented across all students within the sample (‘All students’), and across groups of those with different extents of books at home (where ‘Fewest books’ refers to ‘None or very few (0–10 books)’ and ‘Most books’ refers to ‘Enough to fill three or more bookcases (more than 200)’), and the magnitude (‘D’; Cohen’s D) and statistical significance (‘Sig. (p)’; p-value) of the difference across those with different extents of books at home. Significant p-values (p < .05) and the associated magnitudes are highlighted in bold and shaded. Table adapted from a submission to Open Environment (paper under review).

I have learnt about climate change and/or sustainability…		Comparison Percent who answered ‘*yes*’			
	All students	Most books	Fewest books	Cohen’s d	Sig. (p)
During primary school	77%	80%	72%	**.187**	**.011**
During secondary school	92%	92%	88%	.126	.090
From news and media	78%	83%	67%	**.371**	**<.001**
From my family	61%	78%	36%	**.960**	**<.001**
Through doing activities outside of school, such as through clubs, volunteering, or School Strikes for Climate	43%	49%	30%	**.409**	**<.001**

Table S5:

Percentage of students who indicated that they '*Agree*' or '*Strongly agree*' (rather than '*Neither agree nor disagree'*, '*Disagree*', or '*Strongly disagree*') to seven statements relating to their climate/sustainability education. The results are presented across all students within the sample ('All students'), and across groups of those with different extents of books at home (where 'Fewest books' refers to 'None or very few (0–10 books)' and 'Most books' refers to 'Enough to fill three or more bookcases (more than 200)'), and the magnitude ('D'; Cohen's D) and statistical significance ('Sig. (p)'; p-value) of the difference across those with different extents of books at home. Significant p-values (p < .05) and the associated magnitudes are highlighted in bold and shaded.

To what extent do you agree with the following statements?		Comparison Percent of students who '*Agreed*' or '*Strongly agreed*' (rather than '*Neither agreed nor disagreed*', '*Disagreed*', or '*Strongly disagreed*')			
	All students	Most books	Fewest books	Cohen's d	Sig. (p)
I am able to influence how my school is responding to climate change and sustainability	29%	30%	21%	**.226**	**.002**
Teachers listen to me when I share my views about climate change and sustainability	49%	50%	42%	**.168**	**.021**
My teachers explain how climate change and sustainability are relevant to me and what I can do to make decisions that are more sustainable	60%	62%	47%	**.308**	**<.001**
My teachers explain how climate change can impact different people in different ways across the world	64%	64%	53%	**.214**	**.004**
My teachers help me understand how problems like climate change and the loss of natural environments can be tackled	58%	57%	47%	**.200**	**.006**
I would like to learn more about climate change and sustainability in school	42%	48%	27%	**.442**	**<.001**
I enjoy learning about climate change and sustainability	46%	54%	30%	**.488**	**<.001**

Table S6:

Percentage of students with the most and fewest books at home who indicated that '*Yes, and I HAVE taken part*' to the question '*Do you have the opportunity to take part in any of the following activities in relation to climate change and/or sustainability?*' (rather than '*Yes, but I HAVE NOT taken part*' or '*No*'). The results are presented across groups of those with different extents of books at home (where 'Fewest books' refers to 'None or very few (0–10 books)' and 'Most books' refers to 'Enough to fill three or more bookcases (more than 200)'), and the magnitude ('D'; Cohen's D) and statistical significance ('Sig. (p)'; p-value) of the difference across those with different extents of books at home. Significant p-values ($p < .05$) and the associated magnitudes are highlighted in bold and shaded.

Do you have the opportunity to take part in any of the following activities in relation to climate change and/or sustainability?	Comparison Percent who answered *'Yes, and I HAVE taken part'* *(rather than 'Yes, but I HAVE NOT taken part' or 'No')*			
	Most books	Fewest books	Cohen's d	Sig. (p)
Helping your family to be more sustainable at home	62%	29%	**.709**	**<.001**
Raising concerns about climate change publicly	15%	8%	**.216**	**.001**
Whole school projects related to sustainability	36%	20%	**.349**	**<.001**
Projects with your local community	22%	11%	**.313**	**<.001**
The National Education Nature Park	12%	10%	.075	.287
Arts activities	29%	16%	**.301**	**<.001**
Visits to nature outside school	55%	24%	**.431**	**<.001**
Talks from external speakers (not your teachers)	34%	17%	**.407**	**<.001**
Outdoor learning on the school grounds	40%	25%	**.313**	**<.001**
Other	22%	6%	**.454**	**<.001**

Table S7:

Percentage of students with the most and fewest books at home who indicated '*Yes, and I HAVE taken part*' or '*Yes, but I HAVE NOT taken part*' (rather than '*No*') to the question '*Do you have the opportunity to take part in any of the following activities in relation to climate change and/or sustainability?*'. Provides a measure of the availability of these climate/sustainability activities. The results are presented across groups of those with different extents of books at home (where 'Fewest books' refers to 'None or very few (0–10 books)' and 'Most books' refers to 'Enough to fill three or more bookcases (more than 200)'), and the magnitude ('D'; Cohen's D) and statistical significance ('Sig. (p)'; p-value) of the difference across those with different extents of books at home. Significant p-values (p < .05) and the associated magnitudes are highlighted in bold and shaded.

Do you have the opportunity to take part in any of the following activities in relation to climate change and/or sustainability?	Comparison<br>Percent who responded '*Yes, and I HAVE taken part*' or '*Yes, but I HAVE NOT taken part*' (rather than '*No*')			
	Most books	Fewest books	Cohen's d	Sig. (p)
Helping your family to be more sustainable at home	76%	51%	**.554**	**<.001**
Raising concerns about climate change publicly	41%	34%	**.154**	**.031**
Whole school projects related to sustainability	61%	49%	**.261**	**<.001**
Projects with your local community	50%	38%	**.234**	**.001**
The National Education Nature Park	27%	32%	.119	.102
Arts activities	49%	44%	.104	.150
Visits to nature outside school	69%	60%	**.198**	**.007**
Talks from external speakers (not your teachers)	57%	47%	**.198**	**.006**
Outdoor learning on the school grounds	61%	55%	.127	.077
Other	28%	20%	.184	.068

Table S8:

Percentage of students with the most and fewest books at home who indicated '*Yes, and I HAVE taken part*' (rather than '*Yes, but I HAVE NOT taken part*') to the question '*Do you have the opportunity to take part in any of the following activities in relation to climate change and/or sustainability?*' when activities were available. Provides a measure of participation in climate/sustainability activities when they are available (i.e., among students who did not answer "*No*"). The results are presented across groups of those with different extents of books at home (where 'Fewest books' refers to 'None or very few (0–10 books)' and 'Most books' refers to 'Enough to fill three or more bookcases (more than 200)'), and the magnitude ('D'; Cohen's D) and statistical significance ('Sig. (p)'; p-value) of the difference across those with different extents of books at home. Significant p-values ($p < .05$) and the associated magnitudes are highlighted in bold and shaded.

Do you have the opportunity to take part in any of the following activities in relation to climate change and/or sustainability?	Comparison<br>Percent who responded *'Yes, and I HAVE taken part'* *(rather than 'Yes, but I HAVE NOT taken part')*			
	Most books	Fewest books	Cohen's d	Sig. (p)
Helping your family to be more sustainable at home	82%	57%	**.598**	**<.001**
Raising concerns about climate change publicly	36%	23%	**.276**	**.016**
Whole school projects related to sustainability	58%	41%	**.344**	**.001**
Projects with your local community	45%	28%	**.360**	**.001**
The National Education Nature Park	44%	29%	**.304**	**.020**
Arts activities	59%	37%	**.453**	**<.001**
Visits to nature outside school	80%	57%	**.519**	**<.001**
Talks from external speakers (not your teachers)	60%	35%	**.518**	**<.001**
Outdoor learning on the school grounds	64%	45%	**.397**	**<.001**
Other	79%	32%	**1.073**	**<.001**

Table S9:

Preliminary correlational analyses between students' socioeconomic status and a range of measures capturing emotional, cognitive, and behavioural engagement with the climate crisis. The table presents correlation coefficients ('R', R values) and their statistical significance ('Sig. (p)', p values) between the reported number of books at home (1-5, 1='None or very few (0–10 books)' to 5='Enough to fill three or more bookcases (more than 200)') and the reported indicators. Significant p-values (p < .05) and the associated R values are highlighted in bold.

**Indicator (scale)**	**R**	**Sig. (p)**
Climate change makes me feel: Sad (1=Selected)	**.217**	**<.001**
Climate change makes me feel: Anxious (1= Selected)	**.203**	**<.001**
Climate change makes me feel: Angry (1= Selected)	**.199**	**<.001**
Climate change makes me feel: Afraid (1= Selected)	**.176**	**<.001**
Climate change makes me feel: Guilty (1= Selected)	**.117**	**<.001**
Climate change makes me feel: Ashamed (1= Selected)	**.136**	**<.001**
Climate change makes me feel: Helpless (1= Selected)	**.115**	**<.001**
Climate change makes me feel: Powerless (1= Selected)	**.119**	**<.001**
Climate change makes me feel: Other (1= Selected)	.064	.032
Climate change makes me feel: Despair (1= Selected)	**.146**	**<.001**
Climate change makes me feel: Depressed (1= Selected)	**.090**	**<.001**
Climate change makes me feel: Optimistic (1= Selected)	.039	.088
Climate change makes me feel: Empowered (1= Selected)	.034	.140
Climate change makes me feel: Brave (1= Selected)	**-.059**	**.010**
Climate change makes me feel: Uninterested (1= Selected)	**-.159**	**<.001**
Climate change makes me feel: Happy (1= Selected)	**-.133**	**<.001**
Humans are the main cause of recent changes to the climate (1-5, 5=Strongly agree)	**.198**	**<.001**
There is no doubt about the scientific evidence for warming of the climate (1-5, 5=Strongly agree)	**.302**	**<.001**
There has already been nearly 1 degree Celsius of global warming caused by humans (1-5, 5=Strongly agree)	**.200**	**<.001**
Global warming will slow or stop on its own without humans doing anything (1-5, 5=Strongly agree)	**-.231**	**<.001**
The recent rate of global warming is like that which occurred at the end of previous ice ages (1-5, 5=Strongly agree)	.031	.156
Scientists argue a lot about the cause of global warming (1-5, 5=Strongly agree)	-.034	.112
How often do you do the following to look after the environment: Recycle (0-2, 2=Always)	**.237**	**<.001**
How often do you do the following to look after the environment: Walk, cycle or take public transport (0-2, 2=Always)	**.090**	**<.001**

**Indicator (scale)**	**R**	**Sig. (p)**
How often do you do the following to look after the environment: Turn lights off (0-2, 2=Always)	**.178**	**<.001**
How often do you do the following to look after the environment: Turn the tap off while brushing my teeth (0-2, 2=Always)	**.208**	**<.001**
How often do you do the following to look after the environment: Do things in the garden to help the wildlife (0-2, 2=Always)	**.191**	**<.001**
How often do you do the following to look after the environment: Pick up litter (0-2, 2=Always)	**.134**	**<.001**
How often do you do the following to look after the environment: Think about the environment when I buy things (0-2, 2=Always)	**.207**	**<.001**
How often do you do the following to look after the environment: Read about or watch programmes about the environment (0-2, 2=Always)	**.184**	**<.001**
How often do you do the following to look after the environment: Talk to friends and family about looking after the environment (0-2, 2=Always)	**.174**	**<.001**
How often do you do the following to look after the environment: Eat less meat (including becoming vegetarian or vegan) (0-2, 2=Always)	**.155**	**<.001**
How often do you do the following to look after the environment: Something else (0-2, 2=Always)	.093	.008
How often do you do the following to look after the environment: Try and change what groups in my area are doing (0-2, 2=Always)	-.003	.888
How often do you do the following to look after the environment: Try to change what our country's leaders are doing through activism (0-2, 2=Always)	**-.048**	**.047**
I have learnt about climate change and/or sustainability: During secondary school (1=Yes)	.040	.073
I have learnt about climate change and/or sustainability: From news and media (1=Yes)	**.126**	**<.001**
I have learnt about climate change and/or sustainability: During primary school (1=Yes)	**.056**	**.013**
I have learnt about climate change and/or sustainability: From my family (1=Yes)	**.297**	**<.001**
I have learnt about climate change and/or sustainability: Through doing activities outside of school (1=Yes)	**.113**	**<.001**
My teachers explain how climate change can impact different people in different ways across the world (1-5, 5=Strongly agree)	**.066**	**.005**
My teachers explain how climate change and sustainability are relevant to me and what I can do to make decisions that are more sustainable (1-5, 5=Strongly agree)	**.101**	**<.001**
My teachers help me understand how problems like climate change and the loss of natural environments can be tackled (1-5, 5=Strongly agree)	**.064**	**.006**

**Indicator (scale)**	**R**	**Sig. (p)**
I enjoy learning about climate change and sustainability (1-5, 5=Strongly agree)	**.175**	**<.001**
Teachers listen to me when I share my views about climate change and sustainability (1-5, 5=Strongly agree)	**.079**	**.001**
I would like to learn more about climate change and sustainability in school (1-5, 5=Strongly agree)	**.173**	**<.001**
I am able to influence how my school is responding to climate change and sustainability (1-5, 5=Strongly agree)	.040	.086
Participation: Helping your family to be more sustainable at home (1=Have taken part)	**.222**	**<.001**
Participation: Visits to nature outside school (1=Have taken part)	**.141**	**<.001**
Participation: Outdoor learning on the school grounds (1=Have taken part)	**.064**	**.005**
Participation: Whole school projects related to sustainability (1=Have taken part)	**.118**	**<.001**
Participation: Talks from external speakers (not your teachers) (1=Have taken part)	**.130**	**<.001**
Participation: Arts activities (1=Have taken part)	**.078**	**.001**
Participation: Projects with your local community (1=Have taken part)	**.101**	**<.001**
Participation: Other (please add) (1=Have taken part)	**.155**	**<.001**
Participation: Raising concerns about climate change publicly e.g., public speaking, protesting or writing to an MP (1=Have taken part)	**.057**	**.011**
Participation: The National Education Nature Park (1=Have taken part)	.004	.844
